# Implementability of collecting patient-reported outcome data in stroke unit care – a qualitative study

**DOI:** 10.1186/s12913-022-07722-y

**Published:** 2022-03-16

**Authors:** Lisa Lebherz, Elisa Fraune, Götz Thomalla, Marc Frese, Hannes Appelbohm, David Leander Rimmele, Martin Härter, Levente Kriston

**Affiliations:** 1 Department of Medical Psychology, University Medical Centre Hamburg-Eppendorf, Hamburg, Germany; 2 Department of Neurology, University Medical Centre Hamburg-Eppendorf, Hamburg, Germany; 3 Office for Quality Management and Clinical Process Management, University Medical Centre Hamburg-Eppendorf, Hamburg, Germany

**Keywords:** Implementation, Qualitative methods, Patient-reported outcome measures, Feasibility, Stroke

## Abstract

**Background:**

Patient-reported outcome measures (PROMs) assess patient-relevant effects of medical treatments. We aimed to evaluate the implementation of the International Consortium for Health Outcomes Measurement Standard Set for Stroke (ICHOM-SSS) into routine inpatient care of a stroke unit.

**Methods:**

The ICHOM-SSS was administered in a certified stroke unit during and after inpatient care. Semi-structured interviews with medical staff (*n* = 5) and patients or their proxies (*n* = 19) about their experience were audio-recorded and analysed using thematic analyses. Implementation outcomes were chosen in advance and adhered to current standards of implementation science.

**Results:**

Patients perceived the ICHOM-SSS to be relevant and feasible. They reported limited understanding of why the assessment was introduced. The overall acceptance of using PROMs was high. While medical staff, too, perceived the assessment to be appropriate and relevant, their appraisal of feasibility, sustainability, and their acceptance of the implementation were low.

**Conclusions:**

For a sustainable implementation of PROMs in clinical practice, IT resources need to be adapted, medical care needs to be reorganized, and additional clinical resources are required. Future research should investigate benefits of the ICHOM-SSS and a simpler, automated implementation in stroke care.

**Trial registration:**

ClinicalTrials.gov Identifier: NCT03795948, retrospectively registered on 8 January 2019.

**Supplementary Information:**

The online version contains supplementary material available at 10.1186/s12913-022-07722-y.

## Background

In 2017, over 400,000 inpatients were treated for acute central ischemic or cerebral hemorraghic events in German medical centres [1]. This makes about 2 % of all hospital discharges that year. To date, disability and current risk for secondary cerebro-vascular events in stroke patients are evaluated by standardised external rating scales as well as somatic risk markers (e.g., blood pressure, medical history). Complementary to these measures, the use of additional patient-reported information can capture a more comprehensive picture of the patients’ health and functional status [2, 3]. Prominent patient-reported outcomes (PROs) include mental health symptoms, health-related physical, mental, and social functioning, and quality of life [4].

The inclusion of PROMs to evaluate therapeutic interventions is continuously growing [5], also for cerebro-vascular events [6]. The International Consortium for Health Outcome Measures (ICHOM), an inter-professional non-governmental-organisation, develops standardised and multifactorial questionnaires for the most common conditions with input from patients’ representatives. Its Standard Set for Stroke (ICHOM-SSS) comprises a lean combination of clinical data, administrative data, and PROMs that is suggested for long-term use [7, 8]. Experience of barriers and facilitators of its use in routine stroke care should help to understand necessary circumstances for a successful implementation [9]. This can ultimately lead to increased application of PROMs and a more patient-centred care [10].

**Fig. 1:**
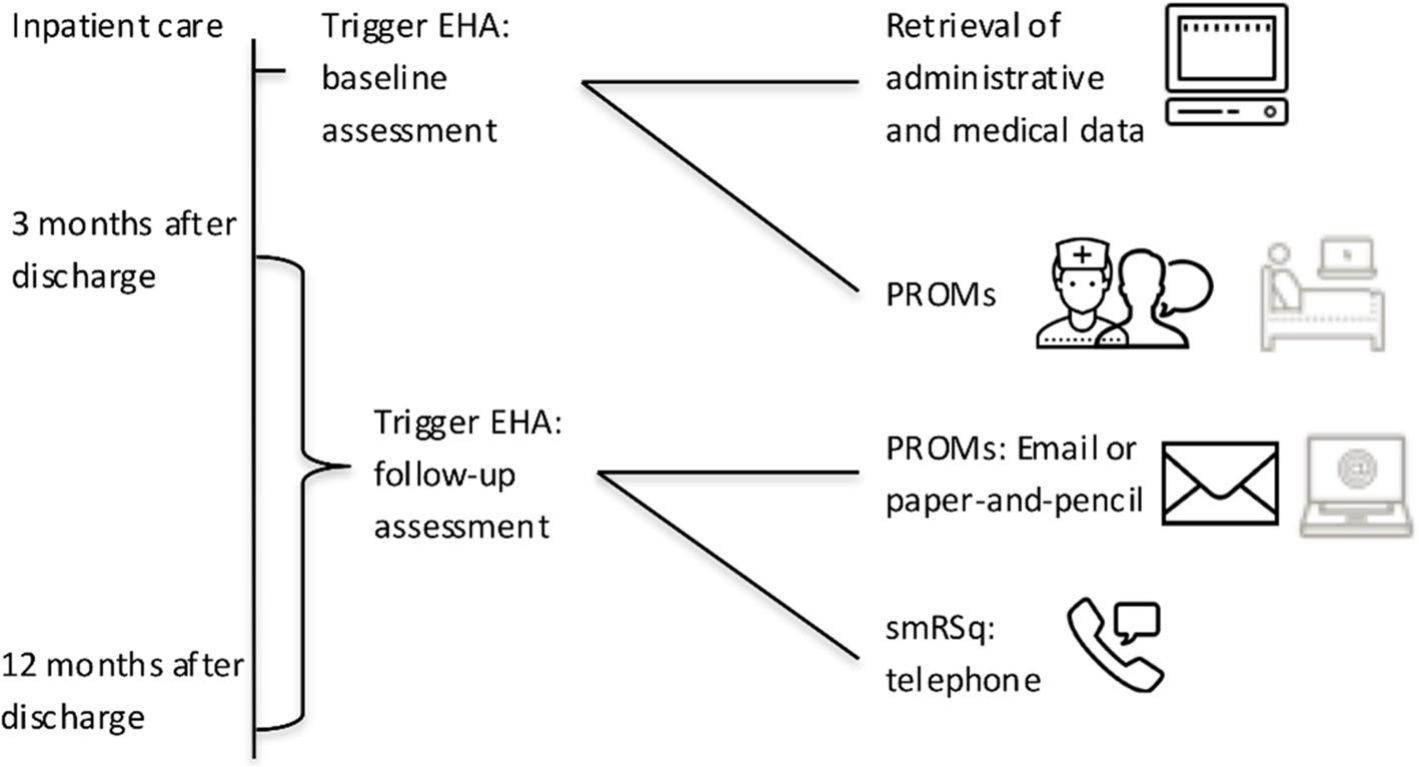
Procedure of the evaluated intervention. The shaded icons denote planned but not realised procedures, see results and discussion. The icons were retrieved from https://icons8.com; EHA = Electronic Health Record, PROM = Patient-reported outcome measure

In a current pilot study, we introduced an ICHOM-SSS-based assessment into routine stroke inpatient care [11]. As the use of pre-existing IT systems facilitates implementation [12], the ICHOM-SSS was integrated into the electronic health record (EHR). This allowed for automated retrieval of medical and administrative data (Fig. 1). In the pilot study, patients were contacted at their bedside, and three and twelve months after discharge. The patient-reported assessments were performed using paper-and-pencil questionnaires, sent by post for the follow-up assessment. In case of no reply, patients additionally were contacted by telephone. An additional functional status measure, the simplified modified Rankin Scale questionnaire (smRSq) [13], was assessed by telephone only. The patient-reported assessment was planned to become automatised over time and linked to the EHR, using the bedside terminal and email link to an electronic assessment after discharge. See Rimmele et al. (2019) [14] for a detailed description of population, recruitment and data acquisition.

In this qualitative study we aimed to evaluate the implementation process from the perspective of staff members and patients.

## Methods

### Design and setting

As part of a monocentric pilot study [14], we performed semi-structured interviews with patients and staff to evaluate the implementation of the ICHOM assessment in a certified stroke unit at a university medical centre in a northern German metropolitan region.

### Cohort and inclusion criteria

*N* = 30 patient interviews were planned. Expecting a certain amount of non-participation, we contacted *n* = 41 patients. This number was considered adequate for a thorough examination of distinct conceptual categories and their interrelation in a sufficiently diverse patient pool [15]. Inclusion criterion for qualitative patient interviews was a completed ICHOM-SSS assessment.

The interviewers were blind to the patient-reported data and knew only name, age, and one-year smRSq score of the patient. Patient participants received oral information about content, rationale, and usage of the interview material. Patients gave an audio-recorded verbal consent to participate in the telephone interview. Patients had already consented to participate in the pilot study and the few returned follow-up assessments in the pilot study led to the conclusion that a number of patients would simply not return signed consent forms per post.

It had been planned to conduct focus groups with clinical staff of the stroke unit. Since the assessment had not been fully implemented during the study (see results), study-external clinical staff had no insight into its routine execution. Hence, interviews were performed with interprofessional staff who were involved in the planning and/or implementation of the assessment (*n* = 5).

### Procedures

Patients and patient proxies were interviewed by telephone after completion of the one-year follow-up. Staff was interviewed in person, once half a year into, and once after termination of data collection.

Guiding questions were developed within the research team. They were clustered into predefined outcomes and were mainly open-ended.

The interviewers (two psychologists, BSc and MSc respectively) were affiliated to the institution which was responsible for the independent evaluation of the research project. The interviewers were informed about the procedures, but were not directly involved in PROM data collection.

### Outcomes

Qualitative implementation outcomes used in this study were adaption, acceptance, appropriateness, feasibility, and sustainability (Table 1)*.* These were selected from a working taxonomy of outcomes for implementation research [16]. We expected overlap between the implementation outcomes. Other thematic trajectories coming from the intervieews were appreciated.

**Table 1:** Qualitative outcomes for implementation research by Proctor et al. (2011) [16]

Outcome	Definition
*Adaption*	intent and engagement in the realisation of the intervention including necessary adoptions to fit the setting
*Acceptance*	stakeholders’ satisfaction with the implementation of the intervention
*Appropriateness*	perceived relevance of the intervention and its compatibility with the setting
*Feasibility*	the extent of successful execution of the intervention
*Sustainability*	the extent of maintenance of the intervention in the designated setting

### Analysis

**Fig. 2:**
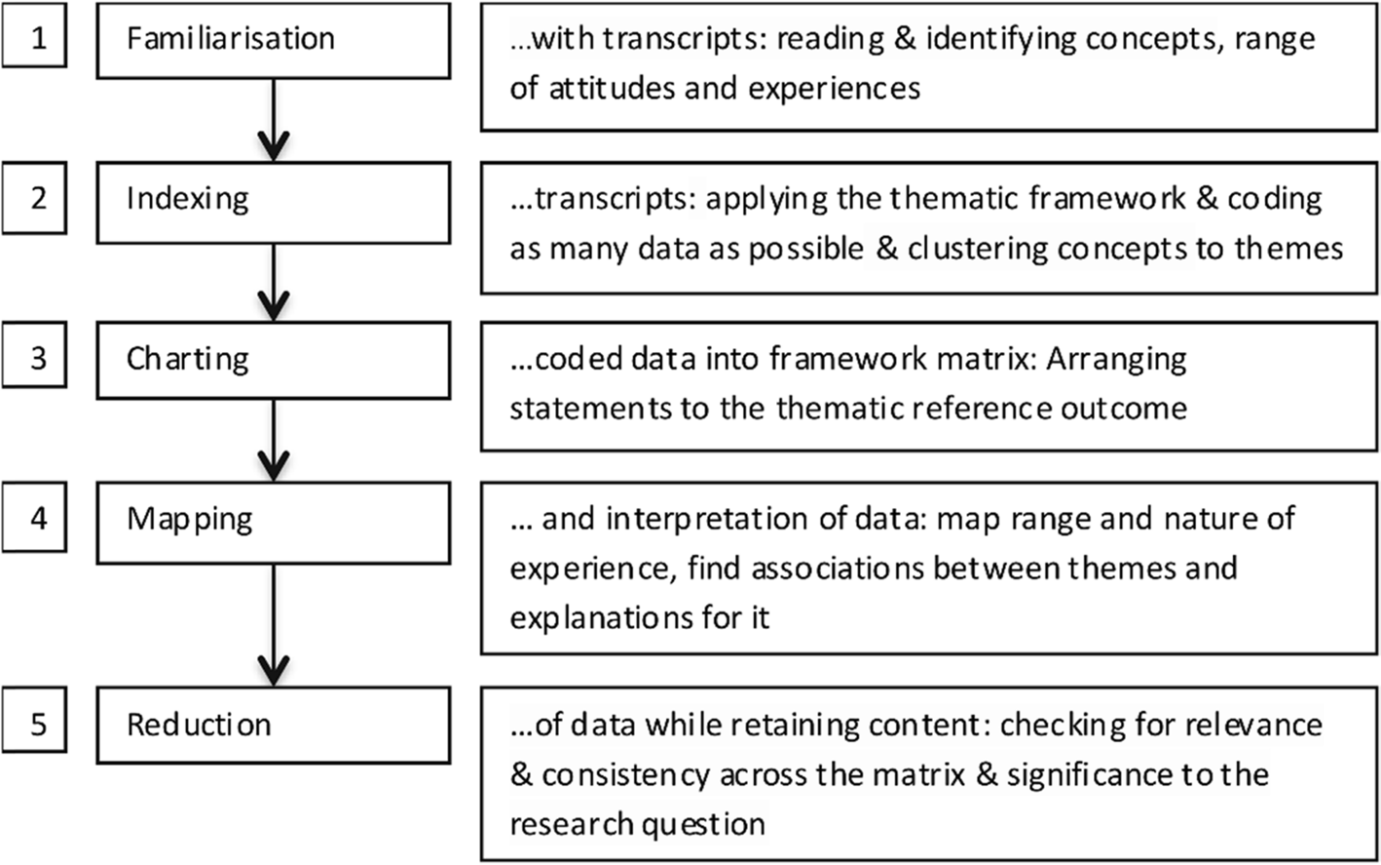
Thematic analysis. We used a mainly deductive framework analysis approach, see Gale et al., (2013) [20]

The interviews were digitally recorded and transcribed afterwards. Guidelines for simple transcription were followed: Nonverbal elements were neglected, and colloquial language was approximated to standard language [17]. The transcripts were imported into MAXQDA (version 10; VERBI GmbH, Berlin, Germany), a computer-assisted qualitative data analysis software package. The analysis followed principles and order of the thematic analysis approach [18] (Fig. 2). The deductive framework for interpretation had been developed before the interviews took place [19]. Themes were pre-defined outcomes (Table 1), although we allowed to create and discuss new categories if adequate [20]. Two researchers independently coded the interviews. Inter-rater consensus was checked by coding a sample of three transcripts that was discussed, refined, and agreed upon. The data was pseudonymised to minimise the possibility to identify subjects and any mentioned third party. Relevant quotes were translated from the German language by the authors. The quotes are organised by superscript numbers. Qualitative data was not quantified [21].

## Results

### Study sample

**Fig. 3:**
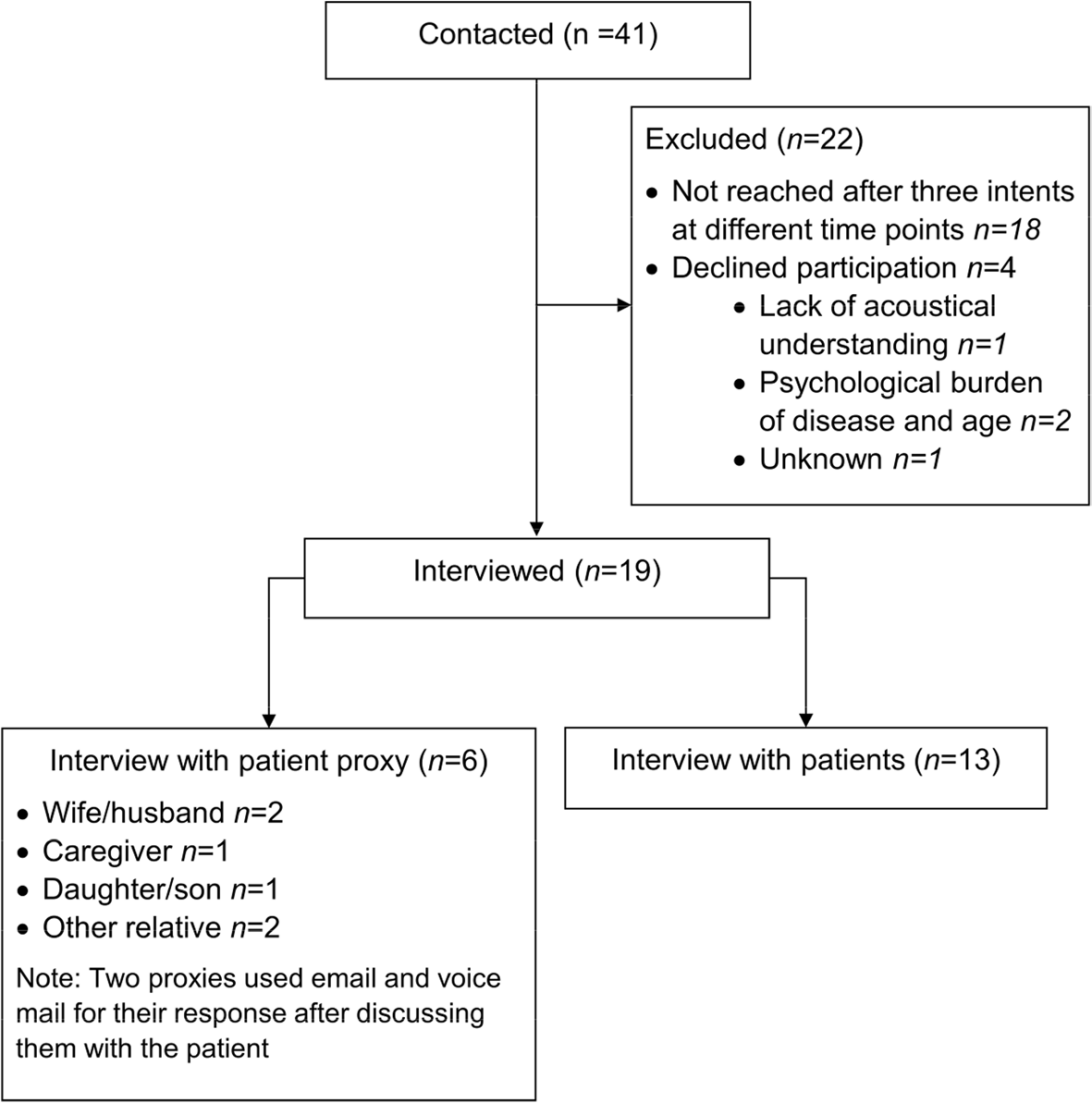
Flow chart of patient and proxy interviews

From the pilot participant cohort, *n* = 41 patients were contacted (Fig. 3 and eFig. 1). Of these, *n* = 13 patients and *n* = 6 patient proxies participated in the interviews. Two patient proxies answered per email and voice mail respectively after discussing the content of the questions with the patient.

The patient collective was a convenience sample. It was selected via quota sampling based on the distribution of smRSq a year after stroke to include a broad spectrum of disease severity (Table 2, eTable 1). Patients whose one-year follow-up was recent to the interview period (September–December 2018) were selected. In cases of severe disability and inability to be interviewed, interviews were conducted with patient proxies.

**Table 2:** Demography of interview partners

Variable	Patient sample *N = 19*	Staff sample *N = 5*
Age, mean (SD)	73.42 (13.9)	
Gender, N (%)		
Female	11 (57.9%)	2 (40%)
Male	8 (42.1%)	3 (60%)
smRSq		
0	4	
1	5	
2	3	
3	3 (3 PP)	
4	2 (1 PP)	
5	2 (2 PP)	
Profession		
Quality Management		1
Medical care		3
IT		1

*SD* Standard deviation, *smRSq* simplified modified Rankin Scale [13] questionnaire, *PP* Patient proxy interviews

Due to saturation of content (i.e., no new aspects and opinions emerged) we decided against replacing non-participants and stopped recruiting after *n* = 19. Patient interviews lasted 03:31 to 14:42 min (median 07:12 min).

Staff interviews (*n* = 5) lasted 14:22 to 84:32 min (median 41:36 min). Nobody rejected participation or was excluded.

**Fig. 4:**
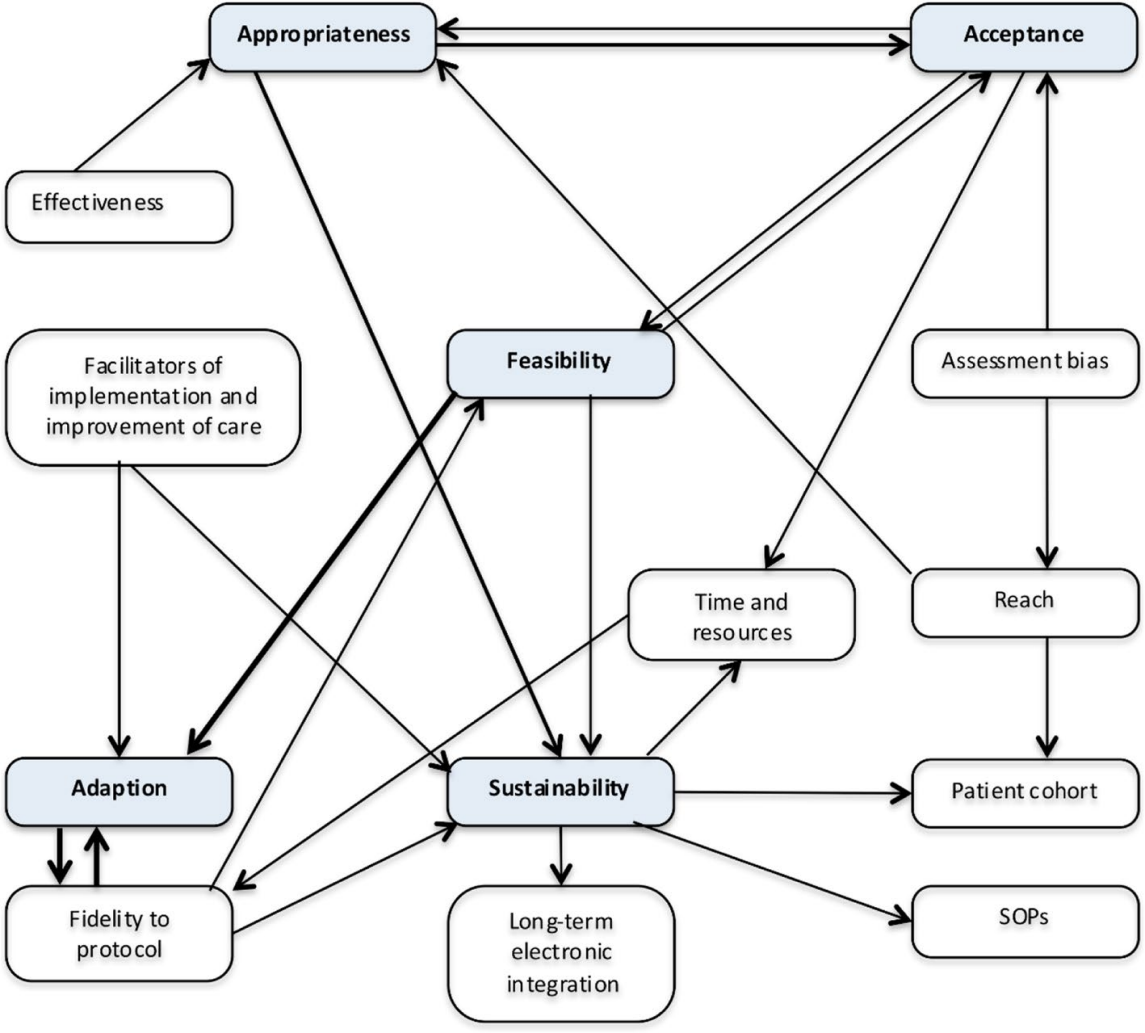
Phase 4 in thematic analysis; schematic model of association between themes and concepts. Arrows indicate expected direction and strength of influence; shades highlight a priori selected outcomes, SOP = standard operating procedure

It was not always possible to allocate a statement to a single implementation outcome (Table 1). Interviewees were likely to refer to several concepts simultaneously. Accordingly, acceptance of staff was strongly determined by (compromised) feasibility. In particular lack of time and resources for proper execution under routine circumstances were determining. Also, it was dependent on anticipated appropriateness and utility (Fig. 4*,* see outcomes below).

### Adaption

The planned smooth transition of the intervention into routine care has not taken place within the study period.

“…*The ‘non-implementability’ without increase in human resources inhibited us.*” ^Q1, staff^

The assessment at inpatient care was done mostly in the presence of or by study staff at the patient’s bedside and in the long-term it was done in paper-and-pencil form that was sent and returned by post.

*“It was planned to have the inpatient questionnaire filled out by the patient alone, but we soon noticed that errors slipped in, e.g., pre-existing conditions were not mentioned or confirmed…”* ^Q2, staff^

*“We could bring a mobile monitor to the hospital bed, so that we could register the answers of the patient directly in the EHR. The disadvantage was, that we were bound to the patient more than we had expected. On the other side, the advantage was that the questionnaire was complete and the patient’s questions could be responded to immediately.”* ^Q3, staff^

Sometimes inpatients were assessed only at discharge rather than at admission (Table 3).

*“You know symptoms from the transfer letter to the stroke unit. And then I know if it is reasonable to survey the patient 24 hours after admission.”* ^Q4, staff^

Due to low response rates of the follow-up questionnaires, the smRSQ telephone assessment was used to supply the follow-up answers directly into the EHR.

**Table 3:** Summary of main themes, source of information and appraisal for each outcome

Outcome	Participant	Appraisal
Acceptance		
Willingness to help scientific advances	Patient	Positive
Fear of giving away information	Patient	Negative
Workload	Staff	Negative
Patient-centeredness	Both	Positive
Appropriateness		
Questionnaire suitable	Both	Positive
Opportunity for better communication	Both	Positive
No recommended actions	Staff	Negative
Feasibility		
Feasible for mildly affected patients	Patient	Positive
Difficult for severely affected patients	Patient	Negative
Feasible to integrate digitally in EHR	Staff	Positive
Not feasible to trigger assessment and retrieve medical data	Staff	Negative
Too much time for nurses	Staff	Negative
Difficult to reach patients in the long-term	Both	Negative
Adaption		
No implementation, for scientific data collection only	Staff	Negative
Adjustments to assessment procedures (baseline and long-term)	Staff	Negative
Sustainability		
SOPs needed	Staff	Negative
Need to simplify and digitalise procedures	Staff	Negative
Leaner assessment needed	Staff	Negative
Software solution needed		
Better retrieval of documentation data needed	Staff	Negative

### Acceptance

Most patients would consent to the ICHOM-SSS assessment again. However, many patients did not understand its patient-centredness with the potential to improve care. Many had participated to help scientific advances and support the medical centre (Table 3).

*“I find it important* […] *because this is a research hospital and therefore you need to do research. That is how I see it. Essentially, you can only improve a patient’s condition with it.”* ^Q5, patient^

However, some patients would not appreciate long-term follow-ups in routine care.

*„...Eventually you must declare, „it’s enough“. People also become older. And imagine* [the patient] *has three other diseases and is to fill in questionnaires for every condition.”* ^Q6, patient^

Some patients were afraid to disclose their data, for fear of losing their statutory nursing care [22] in case of giving “the wrong” answers. A substantial number of patients were challenged with certain questions and procedures. Therefore, many baseline assessments took place as interview by clinical staff rather than in paper-and-pencil form (see adaption). Some patients even criticised this when it was not the case.

*“Maybe it can be discussed in a one-to-one appointment and not via questionnaire.”* ^Q7, patient^

Whereas this negatively affected the acceptance of staff.

*“I had the impression, occasionally patients did not want to respond to certain questions […] like if they drank alcoholic beverages on a daily basis. And if* [the interview] *happens in a room with three patients or relatives sit alongside, that was always problematic.”* ^Q8, staff^

Particularly the inclusion procedure was criticised by staff and acceptance of the procedure was low.

“*I find it quite laborious, we spend an incredible amount of time on the inclusion.”* ^Q9, staff^

Staff’s acceptance towards a general implementation of the ICHOM-SSS was mildly positive, a better realisation provided.

*“Reasonable/ I am absolutely convinced that we need PROMs alongside hard medical outcomes such as mortality.”* ^Q10, staff^

### Appropriateness

Interview partners were satisfied with content, length, and depth of the ICHOM-SSS (Table 3). It was mostly perceived appropriately short. Individual critique concerned depths of content.

*“I had thought, one could work out certain questions much more intensively and introduce additional aspects.”* ^Q11, patient^

Patients and relatives considered such an assessment useful.

*“Well, I really find it highly reasonable. For it doesn’t help if, let’s say, the patient survives but is highly miserable with their fate.”* ^Q12, relative^

Patients with milder symptoms held the assessment rather useful for patients with higher levels of impairment.

*“Maybe one should somehow limit* [the assessment] *to medium to severe cases.”* ^Q13, patient^

Yet, these patients were less likely to report back, especially without external help.

*“It points to a problem, that patients, if I remember correctly, who are affected more severely, are older, more likely to live alone, and are harder to reach.”* ^Q14, staff^

Judgement of appropriateness by staff varied. Some considered PROMs to be highly important in routine care and believed that the ICHOM-SSS was the right tool for it. Others recognised its benefits in its side effects, particularly in extra attendance to patients and the follow-up interviews. Patients used these follow-up calls to update information about their medical condition, which otherwise might not have been addressed in inpatient care or would have become apparent only in the long-term.

*“Depending on the results, I find it highly important that* [implementing the assessment in the routine care] *will be done/ that patients have a contact partner after discharge.”* ^Q15, staff^

Others saw the assessment as reasonable tool to improve treatment quality.

*“We can measure structural quality, we can measure process quality, but we have few instruments to measure treatment quality. And that is why QM* [quality management] *is so interested in PROMs and ICHOM.”* ^Q16, staff^

However, staff was reluctant to define the intervention’s appropriateness before its utility is fully ascertained.

*“Well, when time comes one must look into it: what is the benefit for clinicians?* […] *Is it also suited for individually formed recommended actions?”* ^Q17, staff^

### Feasibility

From the patients’ perspective the assessment was feasible. However, proxy and staff interviews showed the difficulty to collect truly patient-reported information in severe cases.

*“We couldn’t really question him/her, but we have tried to conclude how best to answer the questions from the way we experience him/her. If the patient/ when the disease holds them prisoner so much so that they cannot really respond, then it is difficult.”* ^Q18, relative^

*“But* [it is difficult] *to know if the ones who are truly affected still have quality of life.”* ^Q19, staff^

In this line, staff partly invested disproportionate amounts of time to complete the data and retrieve information from medical reports, e.g., discharge destination (home, or different types of care facilities).

*“You can also ask Google. If you search for a street and see, there is no retirement home, no nursing home but a residential building/ then I can assume that the patient does not live in a care facility but [*lives*] independently. When I don’t find contact information to domestic nursing services, I can assume/ one can draw the conclusion that* [the patient] *does not have a nursing service. But I wouldn’t know 100 per cent.”* ^Q20, staff^

The assessment could be integrated into the EHR with little effort. However, it proved difficult to create an algorithm for a smooth assessment procedure. Staff had to maintain a list with contact details and admission dates to ensure a timely follow-up. Moreover, while an automated follow-up and export of data was planned, raw data had to be requested and processed manually.

*“Actually, the idea was that there was an automatism. When a patient is admitted, the questionnaire is triggered with a to-do list of what needs to be filled out and when. And plainly, that this has not worked out effectively.”* ^Q21, staff^

Staff spent up to 25 min per PROM assessment while it was calculated to take up to 5 minutes. Overlap with other routine interventions (e.g., ward rounds, physiotherapy, medical imaging) and visiting hours made the assessment additionally effortful.

*“The biggest problem is the one of human resources for the administration of PROMs.”* ^Q22, staff^

Staff found that in routine care and by nursing staff alone this procedure and the effort invested to contact patients, to mailing the long-term assessment and to transferring data manually is not manageable.

*“Stroke patients are mostly elderly patients, who are not necessarily connected via mobile phone, who might still be in rehabilitation after three months, or in a nursing home* […] *And if there is no one to make contact with, to call three-four-five times, write letters/… then you won’t find these people anymore. It is again very laborious.“* ^Q23, staff^

### Sustainability

Study staff concluded that the assessment would be implemented properly only, if it were institutionally prescribed (Table 3).

*“Probably a clear decision must be made, usually in form of instructions from above, which makes it clear that this is to be made, this is part of the routine.”* ^Q24, staff^

It was addressed that within the current clinical reality nurses do not have time to spare to assess patient’s well-being beyond what is already done.

*“Calm atmosphere, to sit down, take one’s time, I don’t think so; I don’t believe that nurses can afford to sit on the* [patient’s] *windowsill and drink coffee.”* ^Q25, staff^

It was highlighted repeatedly that solutions must be found to simplify procedures, but foremost that the assessment has to be proven beneficial and appropriate (Fig. 4).

*“*[The assessment method] *must be considerably improved if we want to use it for individual case management or even as a tool to support participatory decision making.’”* ^Q26, staff^

According to staff, ample information from the assessment is already spread in various different locations within the EHR, and at worst, in scans of (handwritten) documents. A more systematic documentation within the EHR was suggested, with more standard answers, (e.g., discharge destination). Also working with text modules would simplify data acquisition.

*“I would say, an additional form would be wrong. It would be more pleasant if the form* [i.e. an algorithm] *retrieved all information from the EHR itself. Or the junction of various forms to one big form that is maintained by physicians and nurses.”* ^Q27, staff^

Staff agreed that a better suited central electronic documentation system is needed to store all information and to visualise data automatically. The idea of using external software was mentioned, including graphical exports of data that could be consulted by clinicians.

*“There must be an automated* [software] *solution. It must be possible to let the majority of patients fill in the questionnaire automatically. And then one would only have to care for a few patients, who cannot complete the assessment by themselves. It must be possible to feed in* […] *and retrieve the data from the EHR automatically. It must be possible that there will be more or less fully automated reports fed back to the clinics.”* ^Q28, staff^

However, complex barriers in the realisation were also seen.

*“For one, the [*external*] connection with the EHR is technically not simple to realise.”* ^Q29, staff^

Accordingly, the fixed-term medical treatment contract would need to be adjusted, so that enquiry of PROs and long-term assessments are permitted without extra requests and patient consent. Currently, the medical centre’s obligation and authorisation to examine the patient ends at their discharge unless the patient consented to participate in registered research that involves long-term assessments.

*“One could imagine that the contract governing medical treatment of the university medical centre shall state that if you do not explicitly object, after certain intervals we will re-examine you* [tele-medically] *because it is our duty/ our aim to monitor outcome quality over the long-term.”* ^Q30, staff^

Staff concluded that the follow-up assessment is not feasible the way it was performed.

*“And then the main issue is/ I believe the data collection from the acute setting is not so bad, but/ the follow-up enquiry, which is not intended in our inpatient health care/. This means one must actively see to get contact information from the patient, must actively approach them and when not reached, repeat contacting or wait that the patient reports back. This is simply not budgeted for.”* ^Q31, staff^

A digital assessment might be practicable, but it was seen problematic to implement in this cohort.

*“Other clinics write emails to patients and have a high return rate. That certainly does not work for stroke patients. It is hence also an issue that lies within the disorder.”* ^Q32, staff^

While patient proxies were less reluctant to use electronic assessment methods patients mostly favoured the paper-and pencil form or interview.

*“I am, as I said, a rather elderly person, so I am a friend of ballpoint and paper.”* ^Q33, patient^

*“We are in the stone ages with*[out] *email, we live in the countryside even without mobile reception in the house.”* ^Q34, relative^

Room for improvement was seen in the communication between treatment facilities and stakeholders.

*What* [the patient] *found lacking were questions about the transition from hospital or rehabilitation facility to community care. From* [the patient’s] *personal experience, there lies considerable need for improvement in the communication between discharging institute and care facility. For one this lack in communication means an overly high effort for us relatives, to organise rehabilitation and medical appointments and the daily routine in the care facility.* ^Q35, relative, answering per email^

## Discussion

This study assessed the implementation of an outcome assessment including PROMs from the perspective of patients and staff in a stroke unit (Table 3).

We could confirm results from studies implementing PROMs in general [23] and the findings of feasibility studies of ICHOM standard sets for other conditions [10]. We found interrelations between outcomes and themes (Fig. 4). Appropriateness mainly mediated the association between acceptance and sustainability. Feasibility and sustainability were dependent on adaption and on acceptance of the concerned parties.

While patients and proxies gave insight into their individual acceptance, perceived appropriateness, and, to a smaller degree, experienced feasibility, staff could elaborate more generally, on more outcomes, and in more detail.

A general introduction of PROMs was seen positive among patients, patient proxies, and staff. The staff considered the effort not justified by the supposed benefit as long as no individualised recommended actions were to follow the assessment. This is in line with a systematic review on PROMs by Boyce, Wick, and Gumbinger (2020) [23], who identified workload associated with collecting and analysing data as a significant barrier for implementation. Other barriers were lack of feedback of results and of impact on care. Defining levels of meaningful change in PROMs should help to establish recommended actions in stroke practice [2].

Studies examining (side) benefits and utility of PROMs demonstrate that their use can positively affect patient-reported health [24]. Measures following PROMs are main contributors to such effects [25]. Also, extra attendance spent on patients, and the feeling of being cared for is perceived relevant for staff and patients [26]. This was confirmed in our interviews. Patients, their proxies, and staff believed that this is particularly important for severely affected patients, while they experienced that these patients were more difficult to reach.

The assessment required substantial personal resources. Documentation of patient response by study nurses was specifically time-consuming. Either dedicated personnel or an automated approach are needed for a successful implementation of PROMs. This is in line with Ackerman et al., (2019) [10] assessing the implementation of an ICHOM standard set for Hip and Knee Osteoarthritis, who’s participants emphasised the importance of a suited IT infrastructure for a sustainable data collection. This would relieve human workload, and make real-time reporting of the data possible [23]. However, our patient population predominantly preferred paper-based versions to an internet-based assessment, while an electronic assessment is more realistically manageable in routine settings.

For a successful implementation, SOPs could regulate the assessment procedure. Studies have registered potentially harmful effects of PROMs, specifically when established in busy practice settings and with low resources and/or acceptance [27]. Thereby, resources allocated to a PRO assessment might lack elsewhere. Careful planning and a suitable infrastructure are essential for successful integration of PROMs. Otherwise, their implementation might add confusion to existing health assessment and decision-making. This was addressed in our interviews.

Gathering missing information from the EHR, as well as the involvement of relatives might compromise assessing PROMs, as data is no longer purely patient-reported [11, 28]. Particularly patients with severe symptoms need external help to complete the assessment. Research has shown that proxies tend to report higher levels of impairment and have difficulties assessing more subjective data [29]. In our interviews, this issue was addressed by patient proxies. In health care settings, especially with disabling diseases such as stroke, missing data is unavoidable [30].

To be an appropriate intervention, the results of a PRO assessment should be available to and used by clinicians to understand the interrelation between disease and experienced health, and most importantly to adjust treatment [31, 32].

### Strengths and limitations

From *n* = 41 contacted patients, only *n* = 13 patients and *n* = 6 proxies participated in the interview (Fig. 3). This cohort was selected from a patient group of the pilot study who completed the one-year follow-up. This might have led to potential bias, as severely affected patients were difficult to reach. However, the smRSq distribution of contacted and participating patient intervieews (or their proxies) was fairly balanced (eTable 1). Also stroke severity (measured with the National Institute of Health Stroke Scale (NIHSS) [33]) was fairly equal among samples (eTable 1).

Patient interviews were very short. Patients had trouble recalling content of the assessment. By reciting items and explaining the rationale behind the interview, answers might have been inadvertently directed.

The interviewers were part of the research team and shared a common goal of implementing the intervention. An interviewer bias is possible.

Interview partners had difficulties to differentiate between intervention and the procedure that was only necessary for scientific evaluation. In this line, the time-consuming study inclusion procedure was a main factor for limited acceptance and implementation. Conversely, extra attendance for patients and their questions was highly valued. However, in a routine implementation it is not realistic to continue such extensive baseline support and contacting patients by telephone after discharge. Furthermore, a substantial number of patients indicated the wish to support medical centre and scientific progress by participating in the pilot study without scrutinising the rationale of PROMs in routine care. While these findings are in line with another ICHOM evaluation [10], they jointly display that low as well as high acceptance of interview partners might be confounded.

Staff interviews were conducted with study staff and not, as planned, with external clinical staff (i.e., staff not involved in the design of the study), because no external staff came in direct contact with the intervention. This might have led to a more positive rating of the intervention. However, besides *acceptance* and perceived *appropriateness* of a general usage of PROMs, the outcomes were mostly negative. Also, three out of five intervieews were clinical staff, who worked in the routine stroke care besides their contribution to the implementation and assessment of PROMs.

Strength of this study were the theoretical basis and the a priori defined domains of interest. Furthermore, patients, health care professionals, and also staff working in IT and quality management were interviewed, so we could generate a broad range of experience with the implementation and contribute to patient-relevant research in the domain of ischemic events.

## Conclusion

In summary, necessary conditions of a successful implementation of the ICOM-SSS and PRO-assessment in general seem to be a supportive IT system integrating and processing long-term PROMs. Also, a less resource-intensive data collection, the help of SOPs regulating the procedures, the possibility to maintain patient contact after discharge, and perceived clinical benefits for the patients facilitate implementation.

## Supplementary Information

**Additional file 1.**

## Data Availability

The qualitative data generated and analysed during the current study are not publicly available as we have not had participants consent to the use of their statements by a third party. However, we are ready to share codebooks and interview guides on reasonable request to the corresponding author.
