# Supplementary material for: Implementability of collecting patient-reported outcome data in stroke unit care – a qualitative study
Source: BMC Health Serv Res. 2022 Mar 16;22:346. doi: 10.1186/s12913-022-07722-y (PMC8925160; doi:10.1186/s12913-022-07722-y)
Supplement: Supplementary file 1 — Additional file 1. [file 12913_2022_7722_MOESM1_ESM.docx]

**Implementability of collecting patient-reported outcome data in stroke unit care – a qualitative study**

Lisa Lebherz^1#^, MSc, Elisa Fraune^1^, BSc, Götz Thomalla^2^, MD, Marc Frese^3^, MBA, Hannes Appelbohm^2^, David Leander Rimmele^2^, MD, Martin Härter^1*^, MD PhD, Levente Kriston^1*^, PhD

**SUPPLEMENTAL MATIERIAL**

eFIGURE 1, Flow chart of patient recruitment and one-year participation in main study


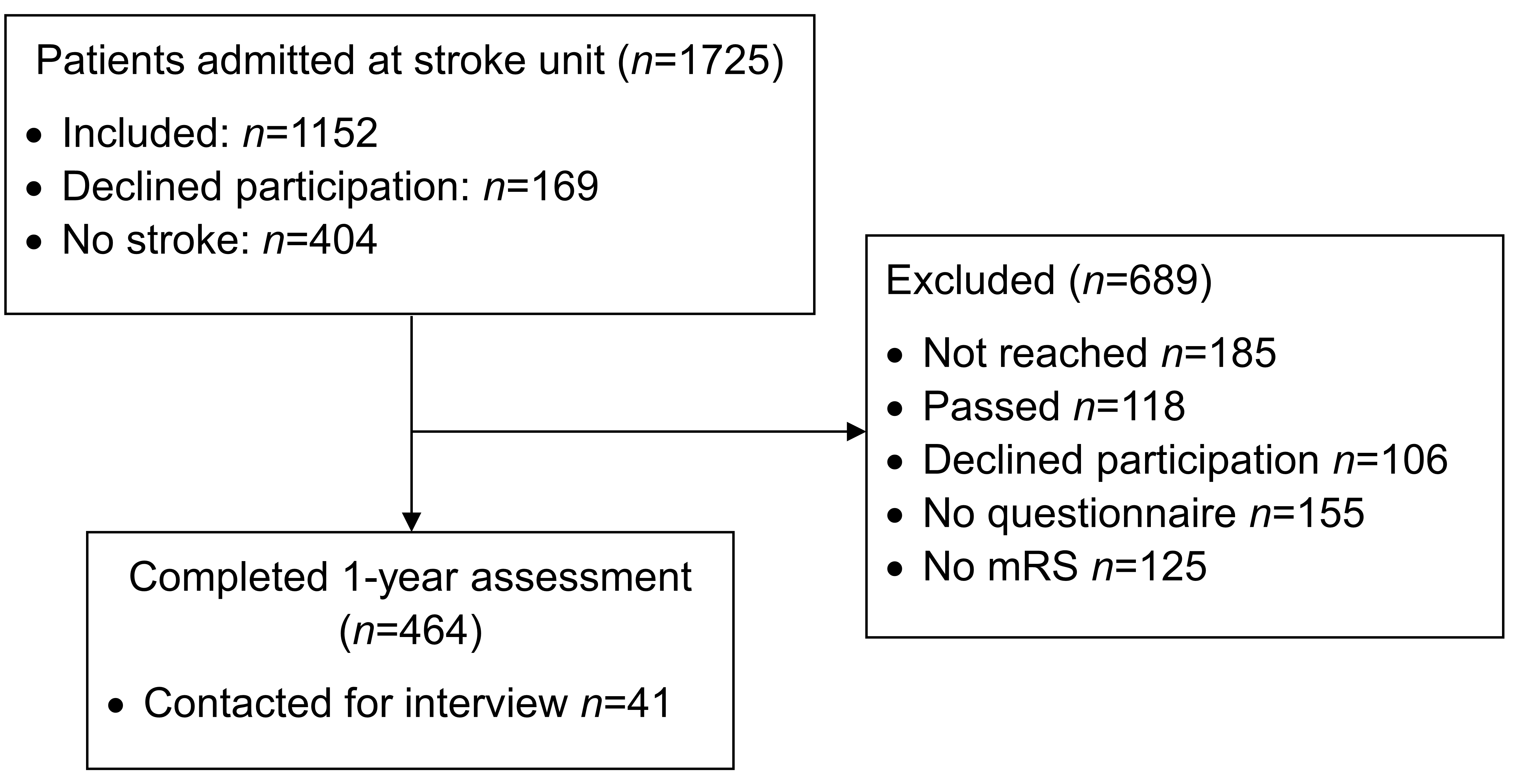


eTABLE 1. Characteristics of various study subsamples

|  | Total pilot sample *N=1152* | One-year responder sample *N=464* | Total interview sample *N=41* | Interview sample *N=19* |
| --- | --- | --- | --- | --- |
| **Variable** |  |  |  |  |
| Age, mean (SD) | 73.62 (12.89) | 73.34 (11.97) | 76.10 (13.24) | 73.42 (13.9) |
| Gender, N (%) |  |  |  |  |
| Female | 557 (48.4%) | 231 (49.8%) | 25 (61%) | 11 (57.9%) |
| Male | 594 (51.6%) | 233 (50.2%) | 16 (39%) | 8 (42.1%) |
| Stroke severity (NIHSS), N (%) |  |  |  |  |
| No stroke symptoms | 324 (28.1%) | 169 (36.4%) | 8 (19.5%) | 5 (26,3%) |
| Mild stroke symptoms | 440 (38.2%) | 177(38.1%) | 21 (51.2%) | 8 (42.1%) |
| Mod. stroke symptoms | 269 (23.4%) | 97 (20.9%) | 10 (24.3%) | 5 (26.3%) |
| Mod. to sev. stroke symptoms | 77 (6.7%) | 17 (3.7%) | 2 (4.9%) | 1 (5.3%) |
| Severe stroke symptoms | 42 (3.6%) | 4 (0.9%) | - | - |
| smRSq |  |  |  |  |
| 0 |  | 192 (41.4%) | 8 (19,5%) | 4 (21.1%) |
| 1 |  | 143 (30.8%) | 11 (26,8%) | 5 (26.3%) |
| 2 |  | 52 (11.2%) | 8 (19,5%) | 3 (15.8%) |
| 3 |  | 45 (9.7%) | 4 (9,8%) | 3 (15.8%) |
| 4 |  | 24 (5.2%) | 6 (14,6%) | 2 (10.5%) |
| 5 |  | 8 (1.7%) | 2 (9,8%) | 2 (10.5%) |
